# Supplementary material for: Tobacco consumption behavior and its associated factors among in-school adolescent students of Saptari, Nepal
Source: PLOS Glob Public Health. 2025 Jan 3;5(1):e0004139. doi: 10.1371/journal.pgph.0004139 (PMC11698322; doi:10.1371/journal.pgph.0004139)
Supplement: Attachment — Submitted filename: Response to Reviewers.docx [file pgph.0004139.s003.docx]

Dear Nnodimele Onuigbo Atulomah, PhD	Date: July 23, 2024
Academic Editor
PLOS Global Public Health

Sir I have tried to address the comments provided by editors and reviewers. What I did to address the comments are present in matrix such as Matrix: Editor Comments and Author Responses, Matrix: Reviewer 1 Comments and Author Responses, Matrix: Reviewer 2 Comments and Author Responses, Matrix: Reviewer 3 Comments and Author Responses. It is attached herewith. Please sir inform me for further needs and requirements. I kindly request you to open my “Supporting Information file” in Statistical Package for the Social Sciences (SPSS).

I am waiting to hear positive from you soon sir.

**Matrix: Editor Comments and Author Responses**

| Editor Comments | Responses of Author |
| --- | --- |
| 1. TITLE: Definitely, the title of the study would required attention. The title appear incomplete because it has not identified the location of the study. 2. ABSTRACT: The abstract needs extensive native English editorial corrections. For example, the first paragraph of the abstract should read: “Tobacco consumption of adolescent students in Nepal have consistently grown to an alarming proportion raising serious concerns of associated factors for the observed trends. This study sought to identify and characterize factors associated with tobacco consumption behavior of these adolescent students.” 3. The abstract is grossly inadequate because the factors of importance and their association with the outcome variable are missing. Therefore, in what way would it be said that this abstract represents the study? 4. Consider appropriate keywords related to MeSh-(Medical Subject Headings). 5. INTRODUCTION: The introduction lacked certain theoretical principles to be resolved based on the statement in lines 62-66. The statement leaves a serious theoretical gap necessary to elucidate observed tobacco consumption among this population. Note that the study is not merely stating the obvious but scientifically substantiating such statement. Without linking this all-important observation to a valid theoretical framework weakens its foundation. Furthermore, how may the observations in lines 238-243 of the study be explained by the existing theoretical framework, apparently there is none, therefore in the absence of any scientifically grounded explanations, the study remains weak, and speculative. 6. METHODS: The section of the methodology needs to be revised to demonstrate precision. For example; line 75: “A cross-sectional survey research design, a non-experimental research where questionnaire or interview is the means of data collection to know the features of population through sample..., was applied for the study.” should be written as “The study adopted a cross-sectional survey design for data collection from consenting adolescents attending the community secondary schools at Hanumannagar Kankalini Municipality, Saptari”. 7. How were the study variables determined without omitting vital other variables. The categorization of the variables appear inappropriate.  The measurement strategies for the behaviour variable is faulty. A re-analysis is required. | 1. I have modified title as “Tobacco consumption behavior and its associated factors among adolescent students of Saptari, Nepal.” 2. I have made slight changes in the sentences related to objectives of the study. 3. Sex of students (P = .001) and education of parents (P = .001) are the factors that associate with tobacco consumption behavior of students. 4. Tobacco consumption behavior of parents is added in the keyword 5. Determinants of tobacco consumption behavior of people such socio-economic (education, occupation, economic condition), demographic (age, sex), spatial (place of residence) and behavioral (tobacco consumption behavior of parents) were identified based on the literatures. Many important variables are left which is limitation of this study. 6. Method section is revised as advised. 7. Tobacco consumed by students ever from the past to the day of the data collection is considered as the tobacco consumption behavior of students. |

**Matrix: Reviewer 1 Comments and Author Responses**

| Reviewer 1 Comments | Response |
| --- | --- |
| 1. There is a need to present the manuscript in a correct and clear English language; you can seek help with some soft applications. From the first sentence in the introduction to almost the entire manuscript, the language lacks clarity, and some sentences are incomplete, some are repetitions. 2. The scope of the study is not completely captured in the title and I suggest the title to be "Tobacco consumption behaviour and associated factors among adolescents", since the study delved deeply into consumption behaviour, in addition to factors associated with them. 3. Results are grossly repeated in the section. Please research what should be contained in the discussion section of a quantitative study. When attempts were made to compare your findings with those of previous studies, they were not properly written and it was difficult to understand the relationship between your findings and those of the cited literature. Thank you | 1. Language of the paper is tried to consider. 2. Title of the study is modified. 3. Some modifications are made in discussion. |

**Matrix: Reviewer 2 Comments and Author Responses**

| Reviewer 2 Comments | Response |
| --- | --- |
| This manuscript presents an investigation to the factors associated with tobacco consumption behavior of adolescent. The manuscript is generally fairly written and clearly presented. Other general and specific comments are provided below: 1. Originality of value: The manuscript presents an original study which was designed and conducted among adolescent students in Nepal. The paper addressed a relevant public health issue of concern, namely tobacco consumption with interesting results. 2. Suitability and soundness of technique: The techniques used for the study are adequate and robust. However, the inferential statistics is inadequate the authors should have utilized binary logistic regression alongside with the chi-square analysis. 3. Clarity of Presentation: The manuscript was fair in narrative presentation. It is easy to follow and complied with the journal specifications. 4. Areas requiring correction. The paper is generally fairly written, there are areas I will like the authors to correct;   1. Title: The title should be change to reflect the study area. Suggested title: Factors associated with tobacco consumption among in-school adolescents in Nepal 2. Abstract   Line 2 on the abstract should not start with although since the consumption of tobacco among the adolescents in Nepal is still a problem. Line 4: the objective of the paper should be written in past tense since authors were reporting their findings. Line 5: the design should simply be written as cross-section design or survey design. Line 7; the authors should change probability proportional to proportional stratified sampling technique. Line 8 instead of stating that the sample size was 235 but only 225 were included in analysis, the authors should state that the response rate was 95.7%. Line 9: the authors need to include that the questionnaire was validated. Line 12: the statistics value to show the association should be included in this abstract. Line 15: the recommendation should be change to be all encompassing not just focusing on the girls and who should be responsible for the recommendation made has to be specified. Line 17 Keywords- factor should change as keywords it can be replaced with parent consumption.   3. Introduction Line 19 and 26 is stating the same thing on the number of death attributed to tobacco. The authors should be mindful of repeating the same thing. The author should clearly state the gap and the uniqueness of the study as the factors were already reported by a study as noted in line 62. 4. Methods Line 75 the authors should state that the study employed a cross-sectional design. Line 102, the authors should explain the basis for different non-response rate. Line 103: the authors should explain in details the sampling technique. How many boys were selected per schools and how many girls? It will be best to have a table to show this distribution and how did they select the students that fill the questionnaire. Line 114: The authors should include where the pretest of instrument was done and the type of validation that was done in details. 5. Results Line 155: the authors should create a table that shows boys and girls separate instead of lumping them together. Line 156: the authors should use a binary logistic regression, Chi-square is not sufficient. Line 256: the recommendation is weak and it should be all encompassing. | 1. Title is modified 2. Line 2 is restructured.   Line 4- objective is written in past tense  Line 5- design was cross-sectional survey design  Line 7- although it was planned to apply probability proportional to size procedure to identify number of students from each school based on identified sample size and number of students admitted in each schools. Then using proportional stratified random sampling to select required number of boys and girls from each schools. But presence of students was not sufficient. So to reach the targeted sample size, all students were participated in the study who were present on the day of study. Description of participated students is present in table 1.  Line 8- is modified  Line 9- validated questionnaire is mentioned  Line 12- statistical value is added  Line 17- factor is removed from keyword and behavior, tobacco consumption behavior of students and tobacco consumption behavior of parents are added  Line 62- issue is mentioned  Line 75- cross-sectional survey design is mentioned  Line 102- basis for non-response rate is mentioned  Line 103- total number of student is mentioned  Line 114- questionnaire was pretested among the 15 students of similar nearby community school of next municipality. Feedback from pretest, discussion with teachers and reviewing literatures were the means to assure validity of tool.  Line 155- number boys and girls are separately presented in table 3 in rows 5 and 6.  Line 156- I have used Chi-square test rather than other test that might be limitation of analysis.  Line 256- recommendation is restated. |

**Matrix: Reviewer 3 Comments and Author Responses**

| Reviewer 3 Comments | Response |
| --- | --- |
| 1. The title syntax requires important modification to enable it to communicate its focus appropriately. The area of focus should be included in the title. Suggestion” Factors associated with tobacco consumption behavior of adolescent student in Saptari, Nepal. 2. Abstract: The abstract requires editorial re-structuring to make it precise. Line 2 should not begin with “although” The description of the methodological approach is not well articulated. Line 5-8 should read thus, “ Cross-sectional survey design was utilized in this study. A sample size of 235 students from community class 10 of Hanumannagar kankalini Municipality, saptari was determined using probability proportion to size method. Statement of conclusion require clarity. 3. Introduction: The introduction requires English language editorials. Line 19-21 can be rephased for clarity of purpose. The deceleration of tobacco cessation program will attribute to 8 million deaths by 2030, where 80% of death will occur in low and middle-income countries. 4. Methods: The statement of the study made in line 75 would be more appropriately expressed: thus The study conducted was a cross sectional survey design to determine Factors associated with tobacco consumption behavior of adolescent student in Saptari, Nepal. The instrument for data collection should be clearly stated. Was the instrument for data collection a questionnaire or interview or both? Tools for data collection; Data collection needs to be detailed. The number of items on the questionnaire needs to be stated and scored. Note; The acronym SPSS should be written in full and line 124 should be corrected “Data analysis was conducted with IBM Statistical Product and Services Solution (SPSS version 20)” 5. Conclusion; Data analysis is flawed and as such discussion of the implications of data attempting to explain the dynamics of the problem phenomenon will be weak and flawed as well. | 1. Title has been modified. 2. Lines 2 and 5-8 are restructured. 3. Lines 19 to 22 are rephrased. 4. Line 75 is rephrased. Anonymous questionnaire was the tool of data collection. IBM statistical package for the social sciences (SPSS) version 20 has been included in the line 124. 5. Discussion part of the paper is modified. |

With Best Regards,

Anil Kumar Mandal

Nepal
