# Supplementary material for: Tobacco consumption behavior and its associated factors among in-school adolescent students of Saptari, Nepal
Source: PLOS Glob Public Health. 2025 Jan 3;5(1):e0004139. doi: 10.1371/journal.pgph.0004139 (PMC11698322; doi:10.1371/journal.pgph.0004139)
Supplement: Attachment — Submitted filename: Response_to_Reviewers_auresp_2.docx [file pgph.0004139.s005.docx]

Dear Nnodimele Onuigbo Atulomah, PhD	Date: Oct 29, 2024
Academic Editor
PLOS Global Public Health

Sir, I have tried to address the comments provided by editors and reviewers, especially Reviewer 2. What I did to address the comments is presented in matrix: Reviewer 2 Comments and Author Responses. Please, sir, inform me about further needs and requirements. Binary logistic regression is used to analyze the data as suggested by Reviewer 2. I kindly request that you open my “Supporting Information file” in Statistical Package for the Social Sciences (SPSS).

I am waiting to hear positive from you soon, sir.

**Matrix: Reviewer 2 Comments and Author Responses**

| **Reviewer 2 Comments** | **Responses** |
| --- | --- |
| **1. Title:** I am suggesting that the author should consider using the term “in-school adolescents” rather than adolescent students  2. **Line 3:** author should rephrased the statement this way: this study describes tobacco consumption behavior of in-school adolescents and its associated factor **3. Line 4:** there is no need to add the phrase for this study: author could simply state that cross-sectional study designed was utilized.  **4. Line 5:** this statement is not clear “Students of community schools of Class 10 of a 6 municipality of Saptari, Nepal were the population of the study”.  **5. Line 6:** this should be written this way; Two hundred and thirty-five students participated in the study with response rate 95.74%  **6. Line 11:** the statistics value to show the association should be included in this abstract.  **7. Line 18:** Keyword: Adolescents, Students, Tobacco, Consumption behaviour  **8. Line 115:** the sampling technique is not clear. How many schools were selected in the municipal? How many students were there in each of the selected schools?  **9. Line 284:** should be captured limitation of study | 1. **Title** is modified by including in-school adolescent 2. **Line 3** is restructured by adding in-school adolescent 3. **Line 4** is modified 4. **Line 5:** a 6 is line number 5. **Line 6** is modified 6. **Line 11:** the statistics values are included 7. **Line 18:** Keywords are slightly modified 8. **Line 115:** I have tried to make it clear by mentioning the number of selected schools and number of students enrolled in each school. 9. **Line 284:** Heading “Limitation of the study” is kept. |

With Best Regards,

Anil Kumar Mandal

Nepal
